# Oil-in-Water Self-Assembled Synthesis of Ag@AgCl Nano-Particles on Flower-like Bi_2_O_2_CO_3_ with Enhanced Visible-Light-Driven Photocatalytic Activity

**DOI:** 10.3390/ma9060486

**Published:** 2016-06-17

**Authors:** Shuanglong Lin, Li Liu, Yinghua Liang, Wenquan Cui, Zisheng Zhang

**Affiliations:** 1 School of Chemical Engineering and Technology, Tianjin University, Tianjin 300072, China; linshuanglong15@126.com (S.L.); chemll@126.com (L.L.); 2 College of Chemical Engineering, Hebei Key Laboratory for Environment Photocatalytic and Electrocatalytic Materials, North China University of Science and Technology, Tangshan 063009, China; liangyh@ncst.edu.com; 3 Department of Chemical and Biological Engineering, University of Ottawa, Ottawa, ON K1N 6N5, Canada

**Keywords:** nanoparticles, oil-in-water self-assembly, Ag@AgCl/Bi_2_O_2_CO_3_, photocatalysis, methylene blue degradation

## Abstract

In this work, a series of novel flower-like Ag@AgCl/Bi_2_O_2_CO_3_ were prepared by simple and feasible oil-in-water self-assembly processes. The phase structures of as-prepared samples were examined by X-ray diffraction (XRD), Scanning electron microscopy (SEM), Transmission electron microscopy (TEM), UV-vis diffuse reflectance spectroscopy (DRS), X-ray fluorescence spectrometer (XRF), *etc*. The characterization results indicated that the presence of Ag@AgCl did not affect the crystal structure, but exerted a great influence on the photocatalytic activity of Bi_2_O_2_CO_3_ and enhanced the absorption band of pure Bi_2_O_2_CO_3_. The photocatalytic activities of the Ag@AgCl/Bi_2_O_2_CO_3_ samples were determined by photocatalytic degradation of methylene blue (MB) under visible light irradiation. The Ag@AgCl (10 wt %)/Bi_2_O_2_CO_3_ composite showed the highest photocatalytic activity, degrading 97.9% MB after irradiation for 20 min, which is over 1.64 and 3.66 times faster than that of pure Ag@AgCl (calculated based on the equivalent Ag@AgCl content in Ag@AgCl (10 wt %)/Bi_2_O_2_CO_3_) and pure Bi_2_O_2_CO_3_, respectively. Bisphenol A (BPA) was also degraded to further prove the degradation ability of Ag@AgCl/Bi_2_O_2_CO_3_. Photocurrent studies indicated that the recombination of photo-generated electron–hole pairs was decreased effectively due to the formation of heterojunctions between flower-like Bi_2_O_2_CO_3_ and Ag@AgCl nanoparticles. Trapping experiments indicated that O_2_^−^, h^+^ and Cl° acted as the main reactive species for MB degradation in the present photocatalytic system. Furthermore, the cycling experiments revealed the good stability of Ag@AgCl/Bi_2_O_2_CO_3_ composites. Based on the above, a photocatalytic mechanism for the degradation of organic compounds over Ag@AgCl/Bi_2_O_2_CO_3_ was proposed.

## 1. Introduction

Bismuth-based oxides with 3D hierarchical architectures have recently attracted much attention among various semiconductor photocatalysts such as Bi_2_O_3_ [1], Bi_2_WO_6_ [2], Bi_2_MoO_6_ [3], BiOCl [4] and Bi_2_O_2_CO_3_ [5] because of their valence bands hybridized by O 2p and Bi 6s [6]. Among these compounds, Bismuth subcarbonate (Bi_2_O_2_CO_3_), as a typical member of the Aurivillius-related oxide family, is constructed by (Bi_2_O_2_)^2+^ slabs interleaved by CO_3_^2−^ layers [7]. Very recently, it has been found to exhibit a promising antibacterial performance and a photocatalytic activity capable of degrading pollutants [8,9,10,11,12,13].

Up until now, various morphologies of Bi_2_O_2_CO_3_ have been obtained. For instance, Zheng and co-workers have synthesized flower-like, sponge-like, porous spheres and plate-like Bi_2_O_2_CO_3_ samples, and have investigated their photocatalytic properties with regards to the degradation of organic pollutants under solar light [14]. They found that the flower-like Bi_2_O_2_CO_3_ assembled by nanoflakes showed the best photocatalytic performance in which over 95% of the RhB solution (10–5 M) was degraded after approximately 50 min. The Bi_2_O_2_CO_3_ microflowers synthesized by Cheng *et al.* could degrade 83% methyl orange (MO) within 150 min, which was much higher than that of commercial Bi_2_O_2_CO_3_ [15].

However, Bi_2_O_2_CO_3_ with the wide band gap can only absorb UV light (less than 5% of the solar light spectrum), restricting its practical application under solar light [16,17]. On the other hand, the photocatalytic efficiency of a photocatalyst is also usually limited by its ability to separate and/or combine photo-generated electron–hole pairs. Therefore, it is necessary to develop an effective strategy to improve charge separation efficiency and to enhance the visible light responsive activity of photocatalysts.

To address this issue, an enormous amount of research effort has been focused on combining Bi_2_O_2_CO_3_ with lower band semiconductors such as MoS_2_ [18], g-C_3_N_4_ [19], Fe_2_O_3_ [20], *etc.* to extend the absorption range. Previous reports revealed that silver@silver halide [21] (Ag@AgX (X = Cl, Br and I)) photocatalysts possess excellent visible light driven photocatalytic degradation efficiencies for organic pollutants and disinfection capabilities due to the localized surface plasmon resonance (SPR) effect exhibited by Ag nanoparticles (NPs). The surface plasmon resonance (SPR) of Ag nanoparticles has been used for photocatalysts due to its excellent absorption in the visible light region. Ag/AgX (X = Cl, Br and I) have become the focus of the photocatalysts due to their extraordinary plasma effect for specific applications, such as visible light harvesting for bacteria destruction and the degradation of organic pollutants in water [22,23]. Importantly, recent works have led to the development of Ag@AgX-based composite photocatalysts with the advantages of efficient visible light absorption, low cost, and relative stability. For example, it has been reported that Ag@AgCl/WO_3_, Ag@AgCl/MCM-41, Ag@AgI/Al_2_O_3_, Ag@AgCl/GO, and AgI/TiO_2_ showed enhanced visible light photocatalytic degradation and disinfection.

To the best of our knowledge, there is no work focused on the preparation of and the photocatalytic activity of Ag@AgCl modified Bi_2_O_2_CO_3_ photocatalysts. Based on previous studies, Bi_2_O_2_CO_3_ modified with Ag@AgCl to form Ag@AgCl/Bi_2_O_2_CO_3_ heterojunctions may improve visible light activated photocatalytic activity. On one hand, 3D hierarchical architectures could make pure Bi_2_O_2_CO_3_ visible light-active through surface scattering and reflecting (SSR) effects [24]. On the other hand, the surface plasmon resonance effects (SPR effects) of Ag@AgCl decorated on the Bi_2_O_2_CO_3_ surface could further strengthen the visible light absorption of 3D Bi_2_O_2_CO_3_ hierarchical microspheres [25]. In this article, Ag@AgCl-decorated Bi_2_O_2_CO_3_ (hereafter designated as NPs Ag@AgCl/Bi_2_O_2_CO_3_) was successfully synthesized via a multistep route, in which a hydrothermal precipitation was employed to synthesize flower-like Bi_2_O_2_CO_3_. An oil-in-water self-assembly synthesis was used to deposit NPs Ag@AgCl on Bi_2_WO_6_. The characterization and a possible photocatalytic mechanism for the Ag@AgCl/Bi_2_O_2_CO_3_ composites were discussed in detail.

## 2. Experimental

### 2.1. Photocatalyst Synthesis

All chemical reagents purchased were of analytical grade and used without further purification. The flower-like Bi_2_O_2_CO_3_ precursor was synthesized by a hydrothermal method. In a typical procedure to prepare flower-like Bi_2_O_2_CO_3_ precursor, 3 mmol of Bi(NO_3_)_3_·5H_2_O were dissolved in 20 mL of 1 M HNO_3_, and then 2 mmol of citric acid were introduced to the solution. After being magnetically stirred for 10 min, the pH of the solution was adjusted to 4–4.2 with the addition of NaOH solution under vigorous stirring. The white precursor that formed was transferred to a Teflon-lined stainless steel autoclave and maintained at 160 °C for 24 h. After cooling the hydrothermal system to room temperature, the flower-like Bi_2_O_2_CO_3_ precursor was separated by a centrifugation process, washed several times with distilled water and ethanol, and dried under vacuum at 80 °C for 8 h.

**Figure 1:**
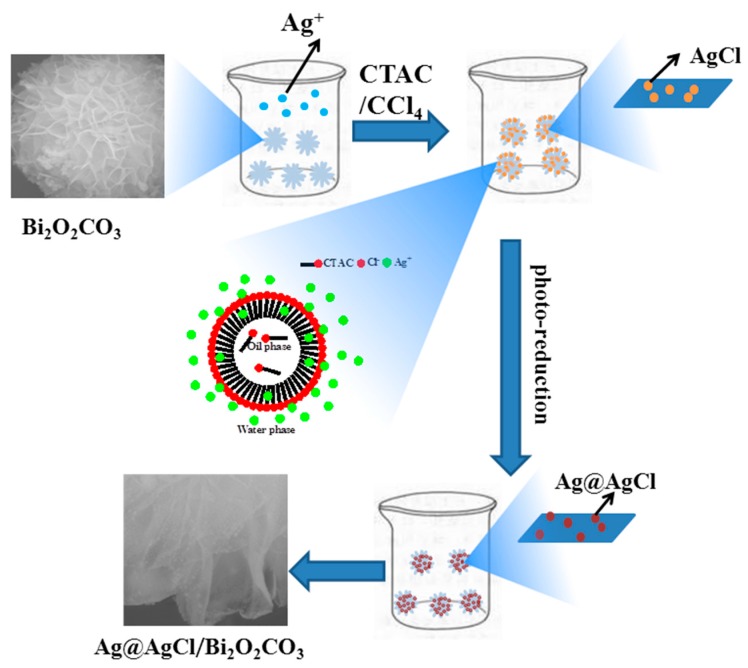
Schematic illustration for the preparation of Ag@AgCl/Bi_2_O_2_CO_3_.

The Ag@AgCl/Bi_2_O_2_CO_3_ composite was prepared by a novel oil-in-water self-assembly method under dark conditions (As shown in Figure 1). Typically, 0.5g Bi_2_O_2_CO_3_ powder and AgNO_3_ were dissolved into 20 mL deionized water and a certain amount (n_AgNO3_:n_CTAC_ = 1:1.5) of Cetyl trimethyl ammonium chloride (CTAC)/carbon tetrachloride solution (40 mL) was added dropwise at room temperature under vigorous magnetic stirring over 20 min. After the CTAC addition, the reaction mixture was magnetically stirred for another 20 min. The resulting suspension was then filtered and washed with deionized water and ethanol, respectively. The obtained AgCl/Bi_2_O_2_CO_3_ powder was dispersed in 30 mL distilled water, and irradiated with a 250 W metal halide lamp (Philips), equipped with wavelength cutoff filters for λ ≥ 420 nm. The resulting sample (Ag@AgCl/Bi_2_O_2_CO_3_) was washed with distilled water and anhydrous ethanol to remove the surfactant, and the final products (hereafter designated Ag@AgCl/Bi_2_O_2_CO_3_) were dried at 80 °C for 8 h in the dark. Ag@AgCl/Bi_2_O_2_CO_3_ composites with different molar ratios of Ag@AgCl to Bi_2_O_2_CO_3_ were prepared according to the typical procedure described above.

For comparison, an Ag@AgCl/Bi_2_O_2_CO_3_ sample was prepared via a precipitation method with Bi_2_O_2_CO_3_ powder, NaOH, AgNO_3_ and CTAC. The as-prepared catalyst was denoted as P-Ag@AgCl/Bi_2_O_2_CO_3_.

Pure Ag@AgCl was synthesized similarly using an oil-in-water self-assembly method with AgNO_3_, and CTAC.

### 2.2. Photocatalyst Characterization

The crystal structures and phase data for the prepared samples were determined by X-ray diffractometry (XRD) using a Rigaku D/MAX2500 PC diffractometer (D/MAX2500 PC, Rigaku Corporation, Tokyo, Japan) with Cu Kα radiation, with an operating voltage of 40 kV and an operating current of 100 mA. The morphologies of the samples were investigated with a scanning electron microscope (SEM) (Hitachi, Chiyoda, Japan, s-4800) and energy dispersive X-ray spectroscopy (EDX), as well as by transmission electron microscopy (TEM) (JEOL Ltd., Akishima, Japan, JEM-2010). UV-visible light (UV-vis) diffuse reflectance spectra were recorded on a UV-vis spectrometer (UV-1901, Puxi, Beijing, China). The chemical compositions of the samples were tested by an X-ray fluorescence spectrometer (XRF, Rigaku, Tokyo, Japan, ZSX Promusll). Surface areas of the samples were determined by the Brunauer-Emmett-Teller (BET) method based on the adsorption and desorption isotherms of N_2_ collected on a Quantachrome Nova 4200e automatic analyzer (Monorosb, Quantachrome, Boynton Beach, FL, USA). The photoluminescence of the powdered samples was measured by a spectrofluorometer (Hitachi, f7000). Electrochemical and photoelectrochemical measurements were performed in a constructed three electrode quartz cell system. A Pt sheet was used as a counter electrode and Hg/Hg_2_Cl_2_/sat. KCl was used as a reference electrode, while the thin film on indium-tin oxide (ITO) was used as the working electrode for investigation. The photoelectrochemical experimental results were recorded with a CHI 660B electrochemical system.

### 2.3. Photocatalytic Activity

The photocatalytic activities of Ag@AgCl/Bi_2_O_2_CO_3_ composites were evaluated by the degradation of methylene blue (MB) under irradiation of visible light. For the first series of tests, a 250 W halide lamp (Royal Philips, Amsterdam, The Netherlands) with a 420 nm cutoff filter was used at a distance of 10 cm from the top of an unsealed beaker. In a second series of tests, a glass reactor with circulating water flowing outside to control the temperature to 25 ± 2 °C was employed. For each test, 0.4 g catalyst powder was added to 100 mL 10 mg/L MB solution. Prior to irradiation, the test solution was stirred in the dark for 30 min. During irradiation, a 3 mL aliquot of the reaction suspension was withdrawn every 5 min and centrifuged at 10,000 rpm for 6 min to remove the particles. The collected supernatant solutions were then analyzed by a UV-vis spectrophotometer.

The degradation efficiency (%) was calculated as follows:

(1) $$D\text{egrada}tion(\%)=\frac{C_{0}-C}{C_{0}}\times 100\%$$

where *C*_0_ is the initial concentration of MB; and *C* is the concentration of MB at time *t*.

Photocatalytic activities while degrading MB in the dark in the presence of the photocatalyst and under visible-light irradiation in the absence of the photocatalyst were also evaluated.

Additionally, the degradation procedure of Bisphenol A (BPA) was the same as that of MB.

## 3. Result and Discussion

### 3.1. Catalyst Characterization

**Figure 2:**
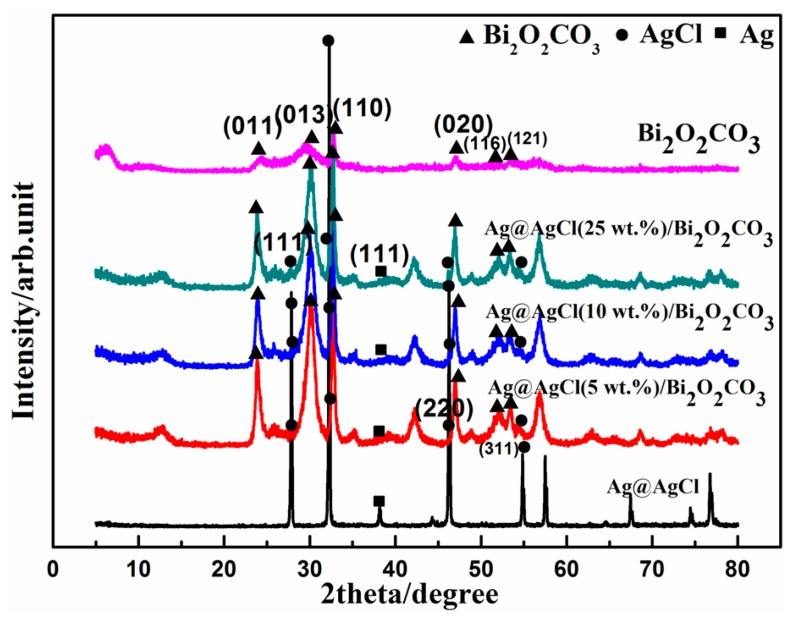
XRD pattern of Ag@AgCl, Bi_2_O_2_CO_3_ and Ag@AgCl/Bi_2_O_2_CO_3_ composites.

The XRD study was carried out to confirm the crystalline structure of the as-prepared Ag@AgCl/Bi_2_O_2_CO_3_ composites. Figure 2 shows the XRD patterns of Ag@AgCl, Bi_2_O_2_CO_3_ and Ag@AgCl/Bi_2_O_2_CO_3_ heterojunctions with different Ag@AgCl contents. The Ag@AgCl was found to be mainly composed of the chlorargyrite AgCl phase. The XRD peaks of AgCl were in good agreement with the cubic crystalline phase of AgCl (JCPDS 31-1238), revealing three distinct diffraction peaks at 27.8°, 32.2°, 46.2° and 54.8°, which can be indexed to the (111), (200), (220) and (311) diffraction planes of AgCl, respectively, while the peaks at 38.1° (111) can be attributed to the small quantity of Ag (JCPDS 04-0783) that formed. The pure Bi_2_O_2_CO_3_ and Ag@AgCl/Bi_2_O_2_CO_3_ both have serious distinct diffraction peaks at the same degree, which can be indexed for bismuth subcarbonate of JCPDS 41-1488. The diffraction peaks observed appearing at 2θ values of 23.9°, 30.3°, 32.7°, 47.0°, 52.2° and 53.4° were assigned to (011), (013), (110), (020), (116) and (121) crystal planes of rhombic Bi_2_O_2_CO_3_, respectively. The presence of Ag@AgCl in Ag@AgCl/Bi_2_O_2_CO_3_ composites was confirmed by the following SEM and TEM images.

**Figure 3:**
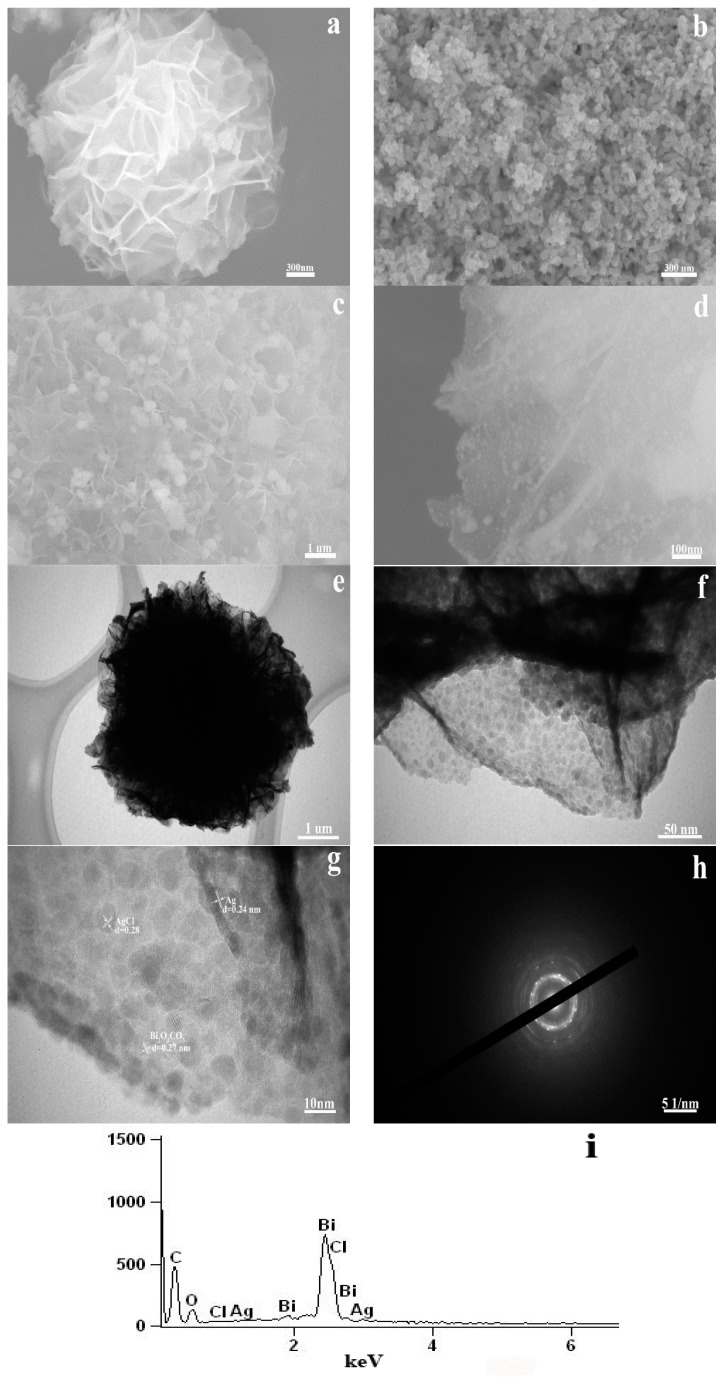
SEM images of: (**a**) Bi_2_O_2_CO_3_; (**b**) Ag@AgCl; (**c**) P-Ag@AgCl/Bi_2_O_2_CO_3_; and (**d**) Ag@AgCl/Bi_2_O_2_CO_3_. TEM images of (**e**,**f**) Ag@AgCl/Bi_2_O_2_CO_3_. HRTEM and SAED images of (**g**,**h**) Ag@AgCl/Bi_2_O_2_CO_3_. EDX spectrum of (**i**) Ag@AgCl/Bi_2_O_2_CO_3_ samples.

The morphology of the Ag@AgCl/Bi_2_O_2_CO_3_ composite was studied by SEM, TEM and HRTEM. It can be observed from Figure 3a that the diameter of a flower-like Bi_2_O_2_CO_3_ microsphere is 1–2 μm, built from two-dimensional nanoplates with a smooth surface. The as-synthesized Ag@AgCl nanoparticles have a nearly spherical shape with a diameter of 80–100 nm (Figure 3b). Figure 3c shows that some Ag@AgCl particles of the size 0.3–0.5 μm are randomly attached to the surface of Bi_2_O_2_CO_3_ microspheres via a precipation method. However, the sample containing Ag@AgCl deposited onto Bi_2_O_2_CO_3_ prepared by a self-assembly method is shown in Figure 3d. The shape of Bi_2_O_2_CO_3_ did not change upon NPs Ag@AgCl-loading, which indicates that the NPs Ag@AgCl deposition process does not damage its flower-like structure. In addition, Ag@AgCl-loading could very well promote the scattering of Ag@AgCl, and reduce its grain size. From Figure 3d, some spherical particles with a size range of 5 to 20 nm were observed to be uniformly deposited on the surface of Bi_2_O_2_CO_3_, indicating the increased surface roughness of the flower-like structure [26]. The BET results revealed that the specific surface area of the Ag@AgCl (25 wt %)/Bi_2_O_2_CO_3_ (24.23 m^2^·g^−1^) nano-composite was larger than that of pure Bi_2_O_2_CO_3_ (13.61 m^2^·g^−1^) (as shown in Table 1). The light absorption was considered to be enhanced due to the increased light scattering. Figure 3e shows the TEM image of flower-like microstructures. According to the TEM image in Figure 3f, Ag@AgCl nanoparticles with a size of about 10 nm were attached to the flower-like nanostructures of Bi_2_O_2_CO_3_. It can be seen from the HRTEM images (Figure 3g), that the clear lattice fringe indicates the high-crystallinity of the composite. The lattice fringes of Ag, AgCl and Bi_2_O_2_CO_3_ coexist in the Ag@AgCl/Bi_2_O_2_CO_3_ composite. The measured interplanar spacings of 0.24 nm, 0.28 nm and 0.27 nm correspond to the (111) plane of Ag, the (200) plane of AgCl and the (110) plane of Bi_2_O_2_CO_3_. This revealed that the nanoparticles formed on Bi_2_O_2_CO_3_ were Ag@AgCl. The SAED pattern collected from Ag@AgCl/Bi_2_O_2_CO_3_ is shown in Figure 3h, where polycrystalline diffraction rings can be observed. The characteristic peak of Ag@AgCl/Bi_2_O_2_CO_3_ can be obviously observed in EDX (Figure 3i). This further proves the formation of Ag@AgCl on Bi_2_O_2_CO_3_. In addition, the elemental compositions of the composite nanoparticles were further analyzed by XRF. The results are shown in Table 2, and the atomic ratio of Ag to Cl was found to be 1.49:1, indicating the presence of Ag in the Ag@AgCl nanojunction system. Based on the XRF results, the atomic ratio of Bi to C to O was found to be 1.9:1:5.5, which was less than the theoretical ratio of 2:1:5 expected from Bi_2_O_2_CO_3_. Since the percentages of O and C were higher in the experimental sample than in the theoretical ratio for Bi_2_O_2_CO_3_, H_2_O and/or C were thought to possibly be present in the prepared Ag@AgCl/Bi_2_O_2_CO_3_ composite.

**Figure 4:**
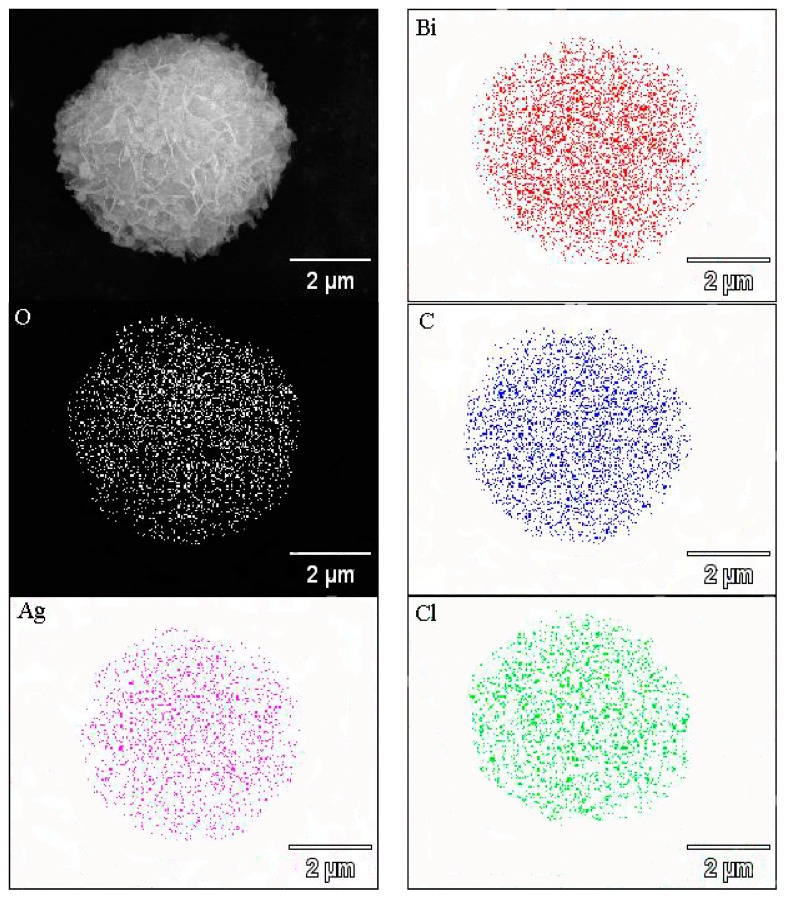
SEM image and EDX elemental maps of spherical structure of Ag@AgCl/Bi_2_O_2_CO_3_ composite photocatalyst.

The EDX elemental mapping of the Ag@AgCl/Bi_2_O_2_CO_3_ composites (Figure 4) displays the even distribution of the elements Ag, Cl, Bi, C and O on Ag@AgCl/Bi_2_O_2_CO_3_. The elements of Ag and Cl are from Ag@AgCl. Bi, C and O are from Bi_2_O_2_CO_3_. The results further indicate the chemical uniformity within individual particles, which clearly confirms that the Ag@AgCl NPs were uniformly distributed on the surface of the Bi_2_O_2_CO_3_ flower-like microspheres. All of the above structure characterizations indicate that a promising Ag@AgCl/Bi_2_O_2_CO_3_ photocatalyst with well-designed architecture was constructed.

**Figure 5:**
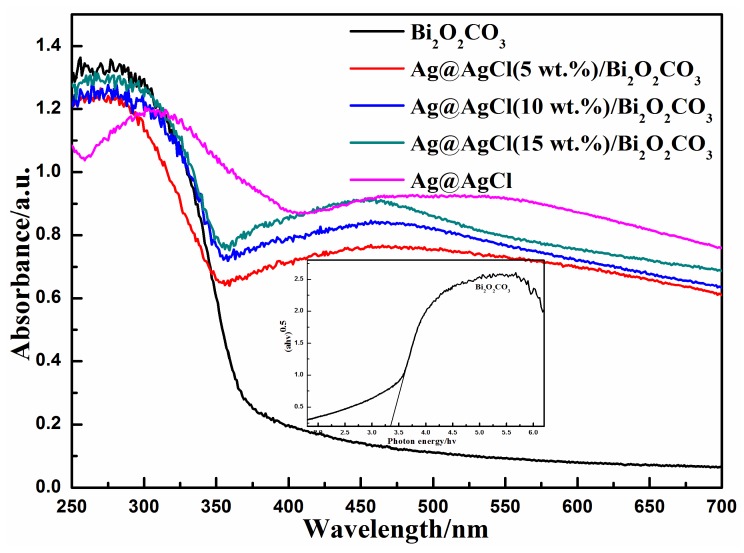
UV-vis diffuse reflectance spectrum (DRS) of as-prepared Bi_2_O_2_CO_3_, Ag@AgCl and a series of Ag@AgCl/Bi_2_O_2_CO_3_ composites (inset: band gap determination of pure Bi_2_O_2_CO_3_ samples).

It is well known that the UV-vis absorption edge is related to the energy band of the semiconductor catalyst, depending on its electronic structure [18]. The optical absorption property of Ag@AgCl/Bi_2_O_2_CO_3_ composites was analyzed. Figure 5 shows the UV-vis diffuse reflectance spectra of Ag@AgCl, Bi_2_O_2_CO_3_ and Ag@AgCl/Bi_2_O_2_CO_3_ composites. Clearly, it can be seen that pure Bi_2_O_2_CO_3_ boasted a weak visible light response with an absorption band edge of about 365 nm. After Ag@AgCl was introduced, the ability of light absorption for Bi_2_O_2_CO_3_ was drastically enhanced due to the surface plasmonic resonance (SPR) of Ag nanoparticles on Ag@AgCl in visible light. Accordingly, with the increase in Ag@AgCl content, the differences in optical adsorption agree well with the colors, changing from white to dark-gray. Additionally, a slight redshift in the absorbance region was observed with the increase in Ag@AgCl content, which might be caused by a possible charge-transfer transition at the interface between Ag@AgCl and Bi_2_O_2_CO_3_, due to the presence of Ag@AgCl [27]. These results also indicate that Ag@AgCl exists on the surface of Bi_2_O_2_CO_3_ and that the Ag@AgCl/Bi_2_O_2_CO_3_ photocatalysts are more effective at degrading organic dyes under light irradiation. On the other hand, for a crystalline semiconductor, the optical absorption near band edge follows the following formula [19]:

(2) αhν=A(hν−Eg)n2

where α, *h*, ν, *E_g_*, and *A* are the absorption coefficient, Plank constant, light frequency, band gap, and a constant, respectively [28]. Among them, *n* is 1 for a direct transition and 4 for an indirect transition. Bi_2_O_2_CO_3_ processes indirect transition band gaps [29,30], and so in this case, the value of n is equal to 4. The *E_g_* value can be estimated by extrapolating the straight portion of the (α*h*ν)^1/2^−(*h*ν) plot to the α = 0 point, where the band gap of Bi_2_O_2_CO_3_ is 3.4 eV (see inset of Figure 5). As a consequence, the Ag@AgCl/Bi_2_O_2_CO_3_ sample can more easily produce electron–hole pairs under the same UV light irradiation and display higher photocatalytic activities.

**Figure 6:**
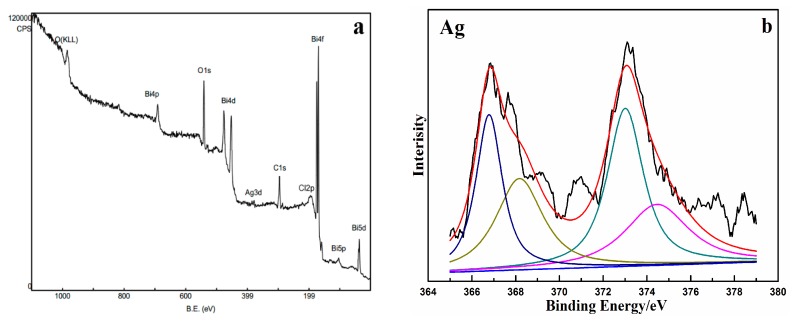
(**a**) XPS survey spectra of Ag@AgCl/Bi_2_O_2_CO_3_; (**b**) High-resolution XPS spectra of Ag 3d.

In order to further determine the chemical state of elements present in the synthesized samples, Ag@AgCl/Bi_2_O_2_CO_3_ samples were analyzed by X-ray photoelectron spectroscopy (XPS), and the results are shown in Figure 6. Figure 6a shows wide-scan XPS spectra of Ag@AgCl/Bi_2_O_2_CO_3_ samples. As shown, the presences of bismuth (Bi 4f, Bi 4d, Bi 4p and Bi 5d), oxygen (O 1s), carbon (C 1s), silver (Ag 3d) and chlorine (Cl 2p) in the Ag@AgCl/Bi_2_O_2_CO_3_ sample were confirmed. Furthermore, high-resolution XPS spectra of Ag in the synthesized sample are shown in Figure 6b. As shown in Figure 6b, for the Ag@AgCl/Bi_2_O_2_CO_3_ sample, the peaks at 373.13 and 366.85 eV correspond to Ag 3d_3/2_ and Ag 3d_5/2_ respectively. It is noted that the Ag 3d_3/2_ peak can be further divided into two separate peaks at 374.46 and 373.01 eV, while the Ag 3d_5/2_ peak can also be divided into two separate peaks at 368.17 and 366.78 eV. The peaks at 366.78 and 373.01 eV can be attributed to Ag^+^ of AgCl, while the peaks at 374.46 and 368.17 eV can be assigned to metallic Ag [31]. The results of XPS analysis confirm the presence of Ag and AgCl in the prepared composite sample.

### 3.2. Photocatalytic Activity

**Figure 7:**
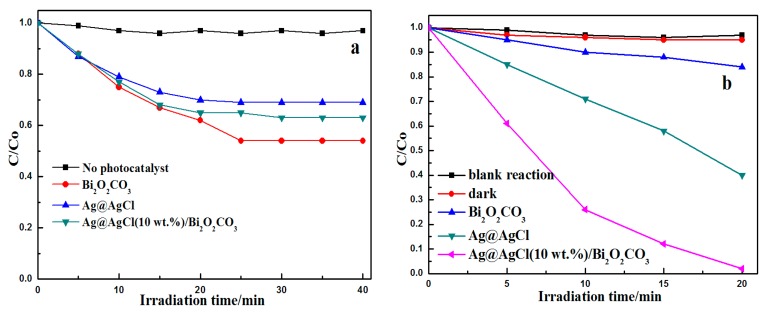
Photocatalytic adsorption (**a**) and degradation (**b**) curves of MB over the various samples.

Figure 7 shows the Photocatalytic adsorption (Figure 7a) and degradation (Figure 7b) efficiency changes in the relative concentration of MB (C/C_0_) as a function of irradiation time over the Ag@AgCl/Bi_2_O_2_CO_3_ composites with components Ag@AgCl and Bi_2_O_2_CO_3_. Figure 7a shows the adsorption performance of Ag@AgCl, Bi_2_O_2_CO_3_ and Ag@AgCl (10 wt %)/Bi_2_O_2_CO_3_. During the dark period, Ag@AgCl, Bi_2_O_2_CO_3_ and Ag@AgCl (10 wt %)/Bi_2_O_2_CO_3_ removed MB from the solution via adsorption. The adsorption equilibrium was reached between 25 and 30 min for these materials. Upon visible light irradiation, as shown in Figure 7b, blank reaction was also given as controls. The blank experiments which were performed in the absence of a photocatalyst showed no obvious changes in the concentration of MB over 20 min of reaction under visible light irradiation. This verifies that MB is an organic dye which is chemically stable and difficult to decompose. The reaction in dark with Ag@AgCl/Bi_2_O_2_CO_3_ catalysts did not facilitate the degradation of MB. After visible light irradiation for 20 min, the degradation percent of MB by pure Bi_2_O_2_CO_3_ was only 26.77%, which indicates that pure Bi_2_O_2_CO_3_ shows weak activity. The degradation of MB in this situation may be induced by the sensitization effects of dye molecules [32], since Bi_2_O_2_CO_3_ cannot be excited by visible light due to its large band gap (3.4 eV). The Ag@AgCl/Bi_2_O_2_CO_3_ composite displayed the highest activity, and the removal percent of MB reached 97.9% within 20 min, which is over 1.64 and 3.66 times faster than that of pure Ag@AgCl (calculated based on the equivalent Ag@AgCl content in Ag@AgCl (10 wt %)/Bi_2_O_2_CO_3_) and pure Bi_2_O_2_CO_3_, respectively. According to these results, the plasmon resonance of Ag NPs and the charge transfer between nano Ag@AgCl and Bi_2_O_2_CO_3_ leads to a higher photocatalytic activity in Ag@AgCl/Bi_2_O_2_CO_3_ compared to the pure Ag@AgCl and Bi_2_O_2_CO_3_ materials.

**Figure 8:**
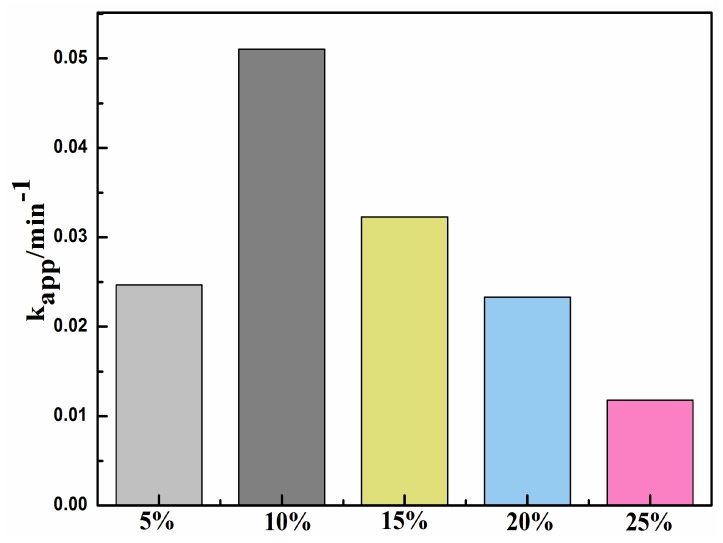
Apparent rate constants for the photocatalytic degradation of MB under the irradiation of visible-light.

The photocatalytic degradation of organic pollutants generally follows pesudo-first-order kinetics. As shown in Figure 8, The k_app_ values of the different samples were calculated in the following order: Ag@AgCl (10 wt %)/Bi_2_O_2_CO_3_ (0.051 min^−1^) > Ag@AgCl (15 wt %)/Bi_2_O_2_CO_3_ (0.032 min^−1^) > Ag@AgCl (5 wt %)/Bi_2_O_2_CO_3_ (0.025 min^−1^) > Ag@AgCl (20 wt %)/Bi_2_O_2_CO_3_ (0.023 min^−1^) > Ag@AgCl (25 wt %)/Bi_2_O_2_CO_3_ (0.012 min^−1^). The Ag@AgCl (10 wt %)/Bi_2_O_2_CO_3_ composite possessed the most optimal activity among all the samples, with an apparent rate constant of 0.051 min^−1^. The highest activity was obtained for the Ag@AgCl (10 wt %)/Bi_2_O_2_CO_3_ sample, on which more than 97.9% of MB was degraded within 20 min. The photocatalytic performance significantly improved after the introduction of Ag@AgCl and increased with the increase of Ag@AgCl content from 0 to 10 wt %. However, further increase in Ag@AgCl loading resulted in a decrease in photocatalytic activity. Once the loading of Ag@AgCl increases beyond a critical value, the nanoclusters of Ag@AgCl species are thought to agglomerate and shade the active sties on the surface of Bi_2_O_2_CO_3_, resulting in lower degradation rates [33]. Simultaneously, this can facilitate the recombination of photoinduced electron–hole pairs. Therefore, suitable Ag@AgCl content was crucial for optimizing the photocatalytic performances of the Ag@AgCl/Bi_2_O_2_CO_3_ composites. The above results imply that significant increases in photoactivity can be ascribed to the synergetic effects of Ag@AgCl and Bi_2_O_2_CO_3_ photocatalyst.

**Figure 9:**
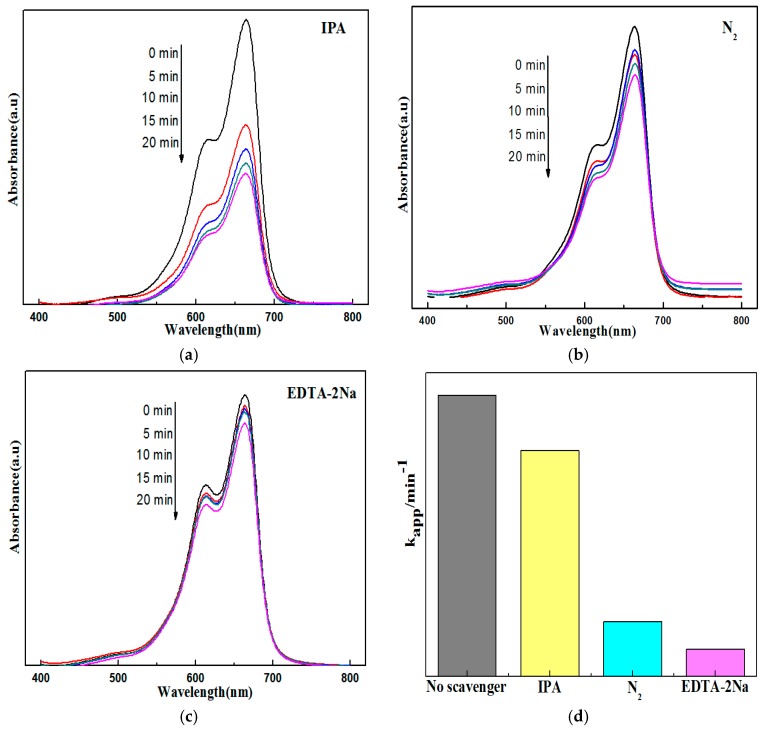
Photocatalytic degradation of MB with Ag@AgCl/Bi_2_O_2_CO_3_ in the presence of various scavengers under visible light. (**a**) IPA; (**b**) N_2_; (**c**) EDTA-2Na; and (**d**)comparison of the visible-light photocatalytic activity obtained using rate constant kapp.

To further ascertain the active species in the degradation process and to verify the supposed photocatalytic mechanism, some sacrificial agents such as tert-butyl alcohol (IPA), ethylenediamine tetraacetic acid disodium salt (EDTA-2Na) and nitrogen (N_2_) were used as the hydroxyl radical (−OH) scavenger, hole (h^+^) scavenger and superoxide radical (−O_2_^−^) scavenger, respectively [34,35]. As shown in Figure 9, the addition of 1 mM IPA produced an insignificant effect on the degradation rate, demonstrating that −OH radicals play a small role in the photocatalytic degradation of MB. In contrast, the photocatalytic degradation rates of MB decreased significantly in the presence of 1 mM EDTA-2Na and under N_2_-saturated conditions. Compared to the addition of EDTA-2Na, N_2_-saturated conditions have a bigger influence, indicating that h^+^ and −O_2_^−^ radicals are the main active species. However, the absolute suppression by IPA, EDTA-2Na and N_2_ on photocatalytic activity was not so large, and so it is thought that another important reactive species may affect the observed activity. Considering that the accumulated holes could oxidize Cl^−^ to Cl°, it was thought that Cl° atoms were the active species that participated in the degradation of MB [36,37]. Accordingly, the active species trapping experiments demonstrated that the h^+^ and −O_2_^−^ are the two main active species in the degradation process of MB, which was also in agreement with the supposed photocatalytic mechanism.

**Figure 10:**
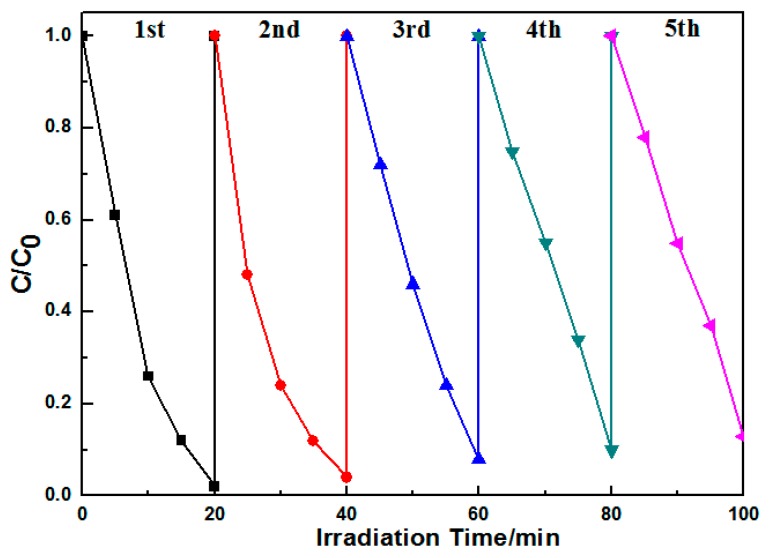
Cycling runs for the photocatalytic degradation of MB in the presence of Ag@AgCl (10 wt %)/Bi_2_O_2_CO_3_ composite under visible light irradiation.

The lifetime of the catalyst is an important parameter of the photocatalytic process. In order to examine the photocatalytic stability of the photocatalyst, the experiment was repeated with the same photocatalyst. The Ag@AgCl/Bi_2_O_2_CO_3_ composite was easily recycled by centrifugation and then washed with water and ethanol. After every 20 min of photocatalytic degradation, the separated photocatalysts were washed with deionized water and then with ethanol to dry them. Figure 10 shows the decrease in MB after every run. A high MB photodegradation could be maintained after five cycling runs with no obvious catalyst deactivation. The slight decrease in photoactivity may be ascribed to the minor loss of catalyst during the recycle experiment [38]. The results confirm that the photoactivity of the Ag@AgCl/Bi_2_O_2_CO_3_ catalyst is stable during the degradation process.

**Figure 11:**
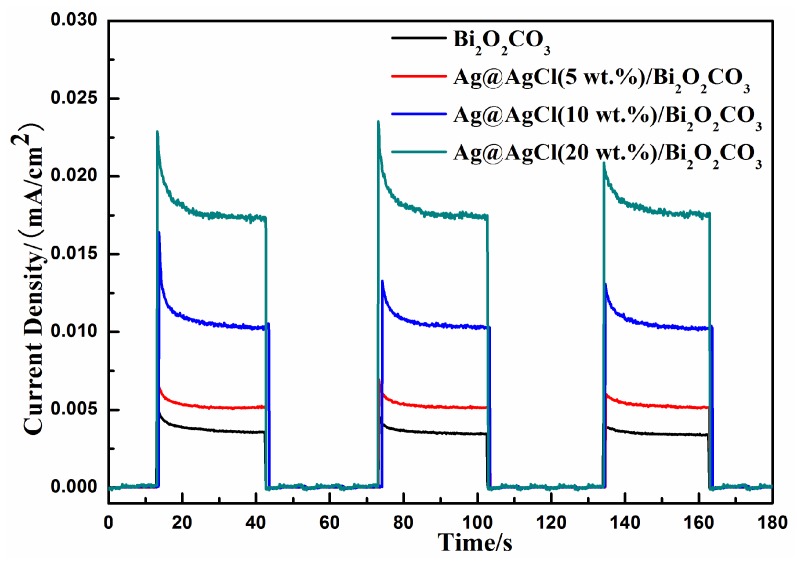
Transient photocurrent response for the pure Bi_2_O_2_CO_3_, Ag@AgCl (5 wt %)/Bi_2_O_2_CO_3_ Ag@AgCl (10 wt %)/Bi_2_O_2_CO_3_ and Ag@AgCl (20 wt %)/Bi_2_O_2_CO_3_.

The interfacial charge separation and transfer dynamics of photoelectrons were studied by monitoring their photocurrent-time responses. As shown in Figure 11, the transient photocurrent responses of Bi_2_O_2_CO_3_ and Ag@AgCl/Bi_2_O_2_CO_3_ were tested and showed three on–off cycles of irradiation. The photocurrent responses of as-prepared samples were obtained by the intermittent visible light irradiation of 30 s. The rise and fall of the photocurrent responses are evidently observed for each switch-on and switch-off event, the results demonstrating effective separation of photoinduced electron–hole pairs in Ag@AgCl/Bi_2_O_2_CO_3_ upon exposure to visible light [39]. The results are shown in Figure 11 (the response of the ITO has been deducted as background). It is clear that Ag@AgCl/Bi_2_O_2_CO_3_ shows a much higher photocurrent density than that of pure Bi_2_O_2_CO_3_. The photocurrent result confirms the more efficient separation of photo-generated electron–hole pairs for the Ag@AgCl/Bi_2_O_2_CO_3_ composite than that of pure Bi_2_O_2_CO_3_. This indicates that Ag/AgCl was been effectively combined with Bi_2_O_2_CO_3_, which leads to the efficient separation of the photo-generated electron–hole pairs in Ag@AgCl/Bi_2_O_2_CO_3_.

**Figure 12:**
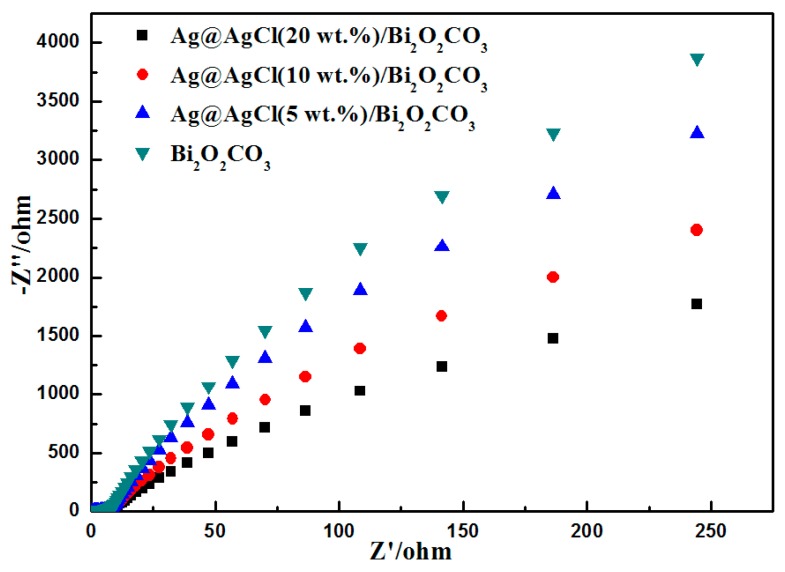
EIS spectra of synthesized Bi_2_O_2_CO_3_, Ag@AgCl (5 wt %)/Bi_2_O_2_CO_3_ Ag@AgCl (10 wt %)/Bi_2_O_2_CO_3_ and Ag@AgCl (20 wt %)/Bi_2_O_2_CO_3_.

Electrochemical impedance spectroscopy (EIS) is a very useful tool to characterize charge-carrier migration [40], and was thus used to further confirm the interfacial charge transfer effects of as-prepared composites. Generally, a higher charge mobility results in a smaller EIS arc radius. Figure 12 shows the EIS Nyquist plots of the Bi_2_O_2_CO_3_ and Ag@AgCl/Bi_2_O_2_CO_3_ electrodes. Obviously, Ag@AgCl/Bi_2_O_2_CO_3_ nanocomposites show much smaller arc radii compared to Bi_2_O_2_CO_3_. The reduced arc radius indicates a diminished resistance of working electrodes, suggesting a decrease in the solid state interface layer resistance and the charge transfer resistance across the solid-liquid junction on the surface by forming a heterojunction between Ag@AgCl and Bi_2_O_2_CO_3_ [41]. Since the radius of the arc on the EIS spectra reflects the migration rate occurring at the surface, this suggests that a more effective separation of photo-generated electron–hole pairs and a faster interfacial charge transfer occurs on the Ag@AgCl/Bi_2_O_2_CO_3_ surface under these conditions [42].

**Figure 13:**
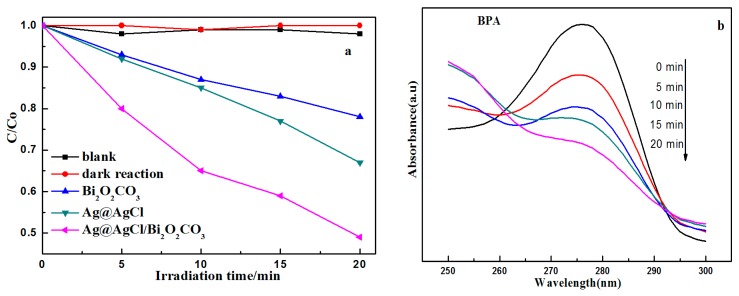
(**a**) Comparison of photocatalytic activities of the as-prepared samples for the photocatalytic degradation of BPA aqueous solutions; and (**b**) UV-vis spectral changes of BPA aqueous solutions in the presence of the Ag@AgCl/Bi_2_O_2_CO_3_ sample under visible light irradiation.

Bisphenol A (BPA) is a colorless organic compound and was chosen to further investigate and compare the photocatalytic activities of Ag@AgCl/Bi_2_O_2_CO_3_ photocatalysts. Temporal changes in the concentration of BPA were monitored by examining the variation in maximal absorption in the UV-vis spectra at a wavelength of 275 nm. Figure 13a shows the photodegradation of BPA (C/C_0_) over Ag@AgCl/Bi_2_O_2_CO_3_ photocatalysts under visible light irradiation. The experimental results obtained indicate that the concentration of BPA was almost unchanged in the dark controls over 20 min, and that BPA molecules are very stable, experiencing almost no decomposition in the absence of a catalyst, which excludes the possibility of photolysis in the present system. The Ag@AgCl/Bi_2_O_2_CO_3_ photocatalyst shows good photocatalytic activity for the degradation of BPA under visible light irradiation compared to Bi_2_O_2_CO_3_ powders and Ag@AgCl photocatalysts. The total photocatalytic degradation of BPA over Ag@AgCl/Bi_2_O_2_CO_3_, Ag@AgCl and Bi_2_O_2_CO_3_ photocatalysts were 50.72%, 32.61% and 21.59%, respectively, after 20 min under visible light irradiation. The UV-vis spectral changes of BPA in aqueous solution over flower-like Ag@AgCl/Bi_2_O_2_CO_3_ photocatalysts under visible light are plotted in Figure 13b as a function of irradiation time. This plot shows that the intensity of the maximum absorption peak of BPA at 275 nm decreases dramatically as visible light irradiation time increases within 20 min.

**Figure 14:**
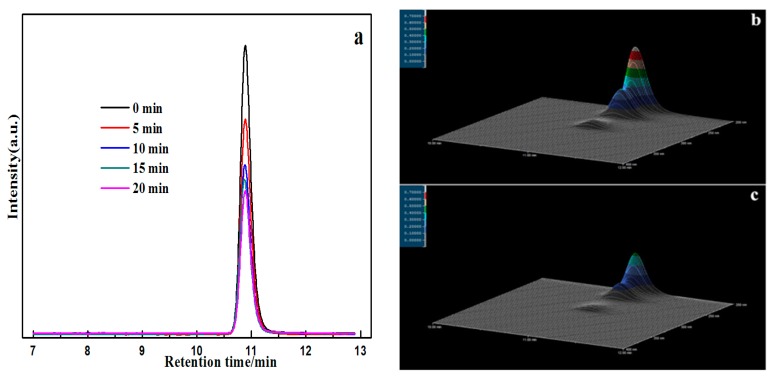
(**a**) HPLC chromatograms of BPA solutions with Ag@AgCl (10 wt %)/Bi_2_O_2_CO_3_ photocatalytic catalyst; (**b**) Three-dimensional HPLC chromatographic spectra of BPA degradation (0 min); (**c**) Three-dimensional HPLC chromatographic spectra of BPA degradation (30 min).

Figure 14a shows the high performance liquid chromatography (HPLC) chromatogram during the photocatalytic degradation of BPA over Ag@AgCl (10 wt %)/Bi_2_O_2_CO_3_ under visible light irradiation. The BPA showed a characteristic peak at a retention time (RT) of 10.9 min. The BPA peak became weaker with increasing irradiation time, and the removal percent of BPA reached 51.4% within 20 min, indicating the excellent photocatalytic activity of Ag@AgCl (10 wt %)/Bi_2_O_2_CO_3_. Three-dimensional HPLC chromatography used to further demonstrate the photocatalytic activity of the Ag@AgCl (10 wt %)/Bi_2_O_2_CO_3_. As the degradation of organic pollutants BPA solution in the reaction of 0 min (Figure 14b) and 20 min (Figure 14c) shown, After 20 min illumination, the BPA corresponding peaks decrease, indicating that BPA was mineralized to CO_2_ and H_2_O in the process of photocatalytic reaction.

**Figure 15:**
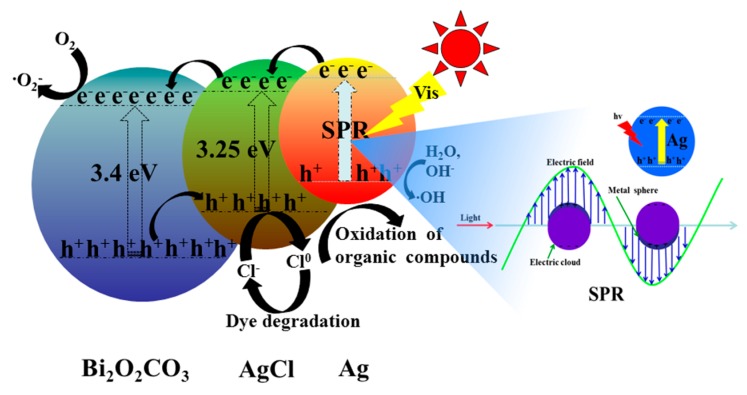
Mechanism of the photocatalytic degradation of MB over Ag@AgCl/Bi_2_O_2_CO_3_.

Combined with the above results, the Ag@AgCl/Bi_2_O_2_CO_3_ composite photocatalyst showed a high photocatalytic activity for the photodegradation of MB dyes under visible light. The possible mechanisms for the enhancement of the photocatalytic activity of the Ag@AgCl/Bi_2_O_2_CO_3_ composite photocatalyst are schematically illustrated in Figure 15. The surface plasmon resonance (SPR) of Ag nanoparticles in the Ag@AgCl/Bi_2_O_2_CO_3_ composite will additionally enhance visible light absorption. Under visible light irradiation, Ag NPs can be excited. Excited state Ag on the catalyst surface can transfer its photo-generated electrons to the CB of AgCl. On the basis of the relative position of the conduction band, the photo-generated electrons will transfer from the CB of AgCl (−0.95 eV) [43] to the CB of Bi_2_O_2_CO_3_ (0.16 eV) [44], making an efficient charge separation and reducing the probability of electron–hole recombination. Simultaneously, the holes in the VB of Bi_2_O_2_CO_3_ can transfer to AgCl, which causes the increased separation rate of photo-generated electrons and holes. The photo-generated holes on the surface of AgCl then react with Cl^−^ to form Cl°. Cl° is a reactive radical species that oxidizes the surface-adsorbed organic pollutants, reducing the Cl° atoms back to Cl^−^. The resultant Cl^−^ reacts with Ag^+^ to form AgCl again, maintaining the stability of the photocatalyst sample under visible light irradiation. While a portion of the holes on the VB of Ag could react with OH^−^ or H_2_O to form hydroxyl radicals (−OH), which have a strong oxidation ability to oxidize organic pollutant molecules, the holes could also oxidize the MB directly [45].

## 4. Conclusions

In summary, a novel flower-like Ag@AgCl/Bi_2_O_2_CO_3_ composite photocatalyst was synthesized by a facile oil-in-water self-assembly method. The introduction of Ag@AgCl nanoparticles into Bi_2_O_2_CO_3_ was found to induce an increased photocatalytic activity under visible light irradiation. The formation of Ag@AgCl/Bi_2_O_2_CO_3_ can improve the optical properties, the surface area, and charge transfer as well as hinder the recombination of electron–hole pairs, which is supposedly linked to the photocatalytic activity. Among the as-prepared samples, Ag@AgCl/Bi_2_O_2_CO_3_ composites with 10 wt % Ag@AgCl showed the highest photocatalytic performances with regards to the degradation of organic pollutants. The photocatalyst also displayed higher stability during the photoreactions and no obvious deactivation was found for the recycled catalyst after five test runs. The present results suggest that hierarchical flower-like Ag@AgCl/Bi_2_O_2_CO_3_ composites are a promising candidate for the photocatalytic degradation of organic dyes.

## Figures and Tables

**Table 1:** Specific surface area of Ag@AgCl/Bi_2_WO_6_ composite catalysts.

Photocatalysts	Bi_2_O_2_CO_3_	Ag@AgCl (5 wt %)/ Bi_2_O_2_CO_3_	Ag@AgCl (20 wt %)/ Bi_2_O_2_CO_3_	Ag@AgCl (25 wt %)/ Bi_2_O_2_CO_3_
Surface area (m^2^·g^−1^)	13.61	14.23	22.07	24.23

**Table 2:** The major elemental content of photocatalysts.

Major Element	Ag	Cl	Bi	C	O
Ag@AgCl (10 wt %)/Bi_2_O_2_CO_3_ (content, mass %)	7.55	1.68	67.28	2.08	16.81
